# Bacterial contamination on used face masks in healthcare personnel

**DOI:** 10.1017/ash.2022.220

**Published:** 2022-05-16

**Authors:** Madison Nightingale, Manali Mody, Alexander Rickard, Marco Cassone

## Abstract

**Background:** Face masks have been worn universally and for long periods of time by healthcare personnel during the COVID-19 pandemic. They are frequently touched or adjusted with the hands and may come in contact with various surfaces and high-touch sites when taken off and on even briefly. These activities present opportunities for face masks to become contaminated with microorganisms. Nursing homes have high rates of multidrug-resistant bacteria and low PPE compliance; therefore, contamination of face masks in this setting may be of great interest. We investigated bacterial colonization status on used face masks in healthcare personnel, including assessing the presence of clinically important and multidrug-resistant bacteria. **Methods:** At a nursing home serving mostly post–acute-care patients, we collected 69 face masks from personnel at the end of the user’s work shift. Information about the mask and the user was also collected via a self-reported survey. Face masks were incubated in BHI broth overnight at 36°C and 10 μL was then plated on selective and differential plates. Methicillin-resistant *Staphylococcus aureus* (MRSA), vancomycin-resistant *Enterococcus* (VRE), and gram-negative bacteria (GNB) resistant to several antibiotic classes were identified using standard microbiological methods. Resistance testing for cefoxitin (*S. aureus*), ciprofloxacin, meropenem, tetracycline, erythromycin, gentamicin, trimethoprim–sulfamethoxazole, and ceftazidime with and without clavulanic acid (gram-negative bacteria) was performed using the disc diffusion technique on Mueller-Hinton plates (Kirby Bauer). **Results:** The job categories of face mask users were competency-evaluated nursing assistant or nursing assistant (22.73%), nurse (12.12%), and other or administrative (37.88%). Overall face mask contamination rates for MRSA (0%) and VRE (3.3%) were low; however, methicillin-susceptible *S. aureus* was found on 11 masks (15.9%). High contamination and resistance rates were found for gram-negative bacteria, with 113 isolates. Among them, 69 (60.9%) were resistant to at least 1 antibiotic, most commonly was erythromycin (59.4%). Additionally, higher rates of clinically important pathogenic gram-negative bacteria were identified: 14.3% of masks were contaminated with *Klebsiella pneumoniae*, 13.0% were contaminated with *Enterobacter* spp, and 4.2% were contaminated with *Escherichia coli*. Importantly, there were no significant differences in the total number of isolates of potential clinical significance recovered from masks worn >6 hours versus those worn <6 hours. **Conclusions:** Among nursing-home healthcare workers, face masks were often contaminated with multiple organisms, including potentially pathogenic bacteria and antibiotic-resistant gram-negative organisms. This contamination may pose a risk for transmission if face masks are not properly used and/or disposed of after wearing. Prolonged duration of face-mask wearing, however, was not associated with increased contamination rates.

**Funding:** None

**Disclosures:** None

